# Ebola in the DRC: A different problem compounded by violence

**DOI:** 10.1371/journal.pntd.0014623

**Published:** 2026-08-07

**Authors:** David M. Brett-Major

**Affiliations:** 1 Department of Epidemiology, College of Public Health, University of Nebraska Medical Center, Omaha, Nebraska, United States of America; 2 Division of Infectious Diseases, Department of Medicine, College of Medicine, University of Nebraska Medical Center, Omaha, Nebraska, United States of America; 3 Global Center for Health Security, University of Nebraska Medical Center, Omaha, Nebraska, United States of America; Institute of Continuing Medical Education of Ioannina, GREECE

## Abstract

A Public Health Emergency of International Concern (PHEIC) has been declared in the Democratic Republic of Congo (DRC) due to Ebola disease from Bundibugyo virus. Non-health-focused factors should be fundamental to how DRC and its partners respond to and recover from the current emergency. Filovirus emergencies are violent in every sense—the disease experience of patients and their caregivers, the tumult in impacted communities, sometimes the physical manifestations of frustration against healthcare services, the fits and starts of concerted response, and the milieu of conflict in which this emergency occurred. Yet, interpersonal and organized violence may contribute to frequency of health emergencies and delays in responding to them. We tend to focus on immediate, direct health needs when managing risks from emerging infectious diseases. Sustained success, though, requires concomitant action on both health and non-health aspects of emergencies and the contexts in which they arise.

The Democratic Republic of Congo (DRC) is one of the world’s top 10 most biodiverse places, the second largest country in Africa in a known endemic zone for several filoviruses [1]. It has experienced at least eight Ebola disease outbreaks in the last 10 years, one of which involved nearly 3,500 patients [2]. This outbreak, however, has been declared a Public Health Emergency of International Concern (PHEIC) [3]. Health emergencies are determined to be a PHEIC when international measures are deemed necessary to limit their spread and consequences. A PHEIC determination is a call to action, either for direct assistance, cooperative measures, or to solicit needed financial or other resources. Now, DRC has experienced two Ebola emergency PHEIC determinations—the current emergency and the 2018–2020 epidemic. The only other filovirus PHEIC was the West Africa 2014–6 epidemic.

By some metrics, that this current emergency is a PHEIC makes complete sense. The emergency was reported to the international community on May 15, and within a week the scale of the emergency was observed to include nearly 800 suspected cases, including almost 180 deaths [4]. As of 17 July, DRC has experienced nearly 2,200 confirmed cases with more than 800 deaths. In ways relevant to a risk equals likelihood and consequence calculation, DRC is distinct from other settings where filovirus emergencies have occurred. Differences between DRC and other countries that have had filovirus emergencies across sub-Saharan Africa can be observed in factors that represent wealth, health and health security commitment, disease burden, and violence (Table 1).

**Table 1:** Comparison of health, wealth, and violence indicators among countries that have had filovirus health emergencies.

Indicator	DRC	Uganda	Rwanda	Guinea	Liberia	Sierra Leone	Nigeria
**Medical doctors**^**α**^ per 10,000 pop.	2.1	2.1	---	1.2	1.8	1.3	4.1
**Health expenditure**^**†**^ per capita (USD)	25	45	53	59	104	35.76	67
**Gross Domestic Product**^**†**^ (USD, M)	70,962	53,912	14,252	25,009	4,779	6,971	252,262
**Homicides**^**α**^ (per 100,000; 2021)	12.4	15.5	4.1	8.5	9.4	7.5	8.8
**Conflict index**^**┴**^ (rank)	High (11)	Turbulent (41)	Low (86)	Low (63)	Low (73)	Low (88)	Extreme (5)
**National institute Nature index** ^‡^	40	114	38	6	6	10	88
**Global Health Security index** ^**¥**^	26	37	33	27	36	33	38
**No. filovirus outbreaks since 2016**	8	4	1	2	1	0	0
**Lag from index case to WHO report (days; most recent event)** ^*^	40–60	10–20	20–30	20–30	---	---	---

^α^World Health Organization Global Health Observatory (https://www.who.int/data/gho); reduced to one decimal place.

^†^From the World Bank, most recent (within last 4 years).

^┴^ACLEDdata.com, for country rank, lower numbers reflect higher conflict activity.

^‡^Overall, by country, https://www.nature.com/nature-index/country-outputs/.

^¥^2021 overall index score at https://ghsindex.org/report-model/; reduced to whole numbers; has a large focus on Joint External Evaluation process and related indicators associated with International Health Regulations (2007) capabilities.

*Estimation from WHO Disease Outbreak News reports based upon each country’s most recent emergency; assumption of 8–10 days interval expansion in similar settings of 1, 8–10, 30, and then onward case counts—only presented for the purpose of comparison and not outbreak characterization; the Guinea 2021 Ebola emergency is represented as a subsequent Marburg outbreak was identified in the midst of escalated response.

In the table, healthcare system indicators are juxtaposed with both interpersonal and organized violence indices; the potential to develop and apply scientific outputs (Nature index, a measure of contributions to the indexed scientific literature from a country); economic indicators; system-level emergency preparedness (Global Health Security Index (GHSI), which assesses progress in achieving International Health Regulations related capabilities); as well as filovirus emergency frequency and time to international reporting. The table only indirectly addresses allied health workforce, infrastructure (e.g., roads and financial systems), and other structural aspects that make each emergency location distinct.

DRC has a relatively high gross domestic product but low investment in health and high rates of interpersonal and organized violence. Broad geographic, biologic, and population diversity coupled with needs at forest interfaces may drive its risks for spillover events, disease moving from animals to people. However, the combination of comparatively high total wealth, scientific productivity, and system-level administrative emergency response mechanisms suggest that DRC could mitigate consequences of spillover events. Its challenges in doing so imply the presence of other factors such as instability that interact with low health investment resulting in more frequent onset of health emergencies as well as delays in recognizing them, and so delays in taking successful cooperative action to end them. That non-health factors could play a role in emerging infectious diseases has been described. Colleagues and I have shown that regardless of the amount of wealth in a country, economic factors are associated with the occurrence of health emergency events [5]. We found that health events were most associated with urban population changes, average forest area, and a novel economic indicator that we developed assessing gross domestic product change per capita. These indicators focused on community-level aspects that drive interactions at the human:animal interface and enable amplification of disease burden. Interpersonal and organized violence can be linked with each of these. In that analysis, Nigeria and DRC led counts of all-cause emerging infectious diseases emergencies. These challenges are not unique to Ebola disease risk management. For instance, DRC remains among the most affected countries in the on-going cholera pandemic [6].

These data flag needs not customarily addressed in conventional health emergency risk management frameworks beyond operational exigencies such as team safety, access, and freedom to operate for social mobilization and intervention [7]. Civil progress is indicated to reduce violence and its triggering and exacerbating factors [8]. When should we focus on such efforts? I posit that it always should be a core focus—it certainly is a core focus of those who must experience violence every day in any community. Civil progress is a preparedness, response, and recovery activity, and yet identification and steps towards addressing long-term objectives in non-health sectors rarely are a part of our exigent assessment and operational focus. DRC has a complex story of multiple interests impacting attainment of peace [9], and among stakeholders (including emergency responders) there may be an unfortunate tendency to presume that current civil status should be assumed to be future status and so not a focus for how risk management of the current health emergency is undertaken. This may increase complacency and widen the possibility that emergency response actions carry unintended consequences.

There are existing structures for coordinating health action in a whole-of-society context. For international actors, that mechanism revolves around diplomacy. In US embassies, the interagency health cluster oriented to international response is customarily led by a professional foreign-service officer. Yet, in my experience, those conversations remain focused on health action and not on how health action may or may not be introducing community harm or promoting civil progress. In health, we typically carry a hierarchy of importance of life, limb, eyesight, and then everything else. This provides clarity, except when attempting to understand ways in which systems that we build to meet those demands impact community function. Ostensibly, that foreign service officer and her counterparts in other countries’ diplomatic missions are working behind the scenes to apply other levers of influence to mitigate consequences or yield opportunity from how we focus our health actions. Or, they simply are grateful for the soft power of health emergency response that is a pillar of how every state and international body attempts to influence local behavior.

In these contexts, one can quickly appreciate the utterly non-aligned approach of Médecins Sans Frontieres (Doctors without Borders). In their and other responders’ positions, detachment is relied upon to avoid harm through an express focus on life. Such work emphasizes the value of people. If it promotes social change such as violence mitigation, it does so through an almost clergy-like long view of promoting humanism.

In health response, the emphasis of discourse and action against violence and for the imperative of safety has focused on healthcare in danger. The International Red Cross leads an important initiative to promote sanctity in seeking and delivering shelter and care. In a coalition with the Red Crescent Movement, they address “the issue of violence against patients, health workers, facilities and vehicles,” a critical effort in an environment of reckless disregard of human connection and intervention [10]. But what of communities in danger? For these response agencies, they are a call for service and concerted action. This meets a critical need, but does not necessarily address recovery into a more resilient future.

In the case of organized violence, participants understand opportunities and risks from health emergencies—influence locally and among those seeking access; resources both against the health threat and divertible purposes; and, both visibility and exposure. The balance of those and whether or not harm is likely due to approaches taken by external responders is difficult to discern when arriving emergently to a community. This is yet another reason why local interests and local leadership are incredibly important to successful health emergency response.

Those connected longitudinally to communities working against Neglected Tropical Disease (NTD) burden have much to offer in relating lessons learned on how best to understand local interests and support those communities usefully. NTD programs often are long-lived and so more visibly intertwined with durable community issues. Such knowledge and access has the potential to enable better identification of stakeholders necessary for a successful response while also helping to inventory interests that must be accounted to ensure greatest benefit and reduced harms to the community. In some areas where I have worked, long-running programs advancing nutrition empowered local leaders to relate preferences and needs, and also helped planning and identification of local surge workforce from those whose day jobs were disrupted by the health emergency. On a larger systems level, there could be a more integrated role for social, political and economic expertise in response structures to widen the scope of such knowledge and its application.

When we make heavy investments to intercede on a health emergency and take our own technical actions in the response [11,12], we also should be asking, what alternative or parallel investments might mitigate the conditions under which the health emergency occurred and are less well controlled? How soon can we start intervening on the course of recovery and resilience through a focus on communities’ durable challenges? Since the lessons that we hold often are from the most recent experiences we have, rigor in assessing our on-going experience and taking rapid steps towards recovery earlier in response activities may prove useful in preventing and mitigating risks from Ebola disease and other threats in the future. In this emergency, we should pay attention to the influences of violence and the need for active diplomacy that looks beyond the current emergency in conjunction with our exigent health-focused interventions.

These views are those of the author and do not necessarily reflect those of any agency.
